# Ovarian Hyperstimulation Syndrome (OHSS) requiring Intensive Care Unit (ICU) admission between 1996-2020 in England, Wales, and Northern Ireland

**DOI:** 10.3389/fendo.2022.1060173

**Published:** 2022-12-15

**Authors:** Ali Abbara, Bijal Patel, Isha Parekh, Akanksha Garg, Channa N. Jayasena, Alexander N. Comninos, Waljit S. Dhillo

**Affiliations:** ^1^ Section of Endocrinology and Investigative Medicine, Imperial College London, Hammersmith Hospital, London, United Kingdom; ^2^ Imperial College Healthcare NHS Trust, Charing Cross Hospital, London, United Kingdom

**Keywords:** Ovarian Hyperstimulation Syndrome (OHSS), *In Vitro* Fertilization (IVF), Intensive Care Unit (ICU), rates, severe OHSS

## Abstract

**Introduction:**

Ovarian Hyperstimulation Syndrome (OHSS) is a life-threatening iatrogenic complication of *In vitro* fertilisation (IVF). This study aimed to quantify rates of Ovarian Hyperstimulation Syndrome (OHSS) requiring intensive care unit (ICU) admission and assess whether trends have changed between 1996-2020 commensurate with the introduction of safer IVF practices.

**Methods:**

Data regarding Intensive Care Unit (ICU) admission across England, Wales and Northern Ireland was gathered retrospectively from the Intensive Care National Audit and Research Centre (ICNARC) database. 38,957 female patients aged between 18-55 years were admitted to ICU for OHSS or related conditions between 1996-2020. The primary outcome was the rate of OHSS requiring ICU admission expressed as a proportion of the number of fresh IVF cycles conducted in that year according to Human Fertility and Embryology Authority (HFEA) records. Baseline characteristics (for example, age, ethnicity, BMI), biochemical parameters (such as renal function, serum electrolytes), length of ICU stay and duration and need for organ support, were also compared between ICU patients with ‘confirmed OHSS’ and those ‘without OHSS’.

**Results:**

There were 238 cases of ‘confirmed OHSS’ requiring ICU admission recorded between 1996-2020. Rates of OHSS requiring ICU admission declined over the study period (P=0.006); the annual rate of severe OHSS requiring intensive care admission halved when comparing those occurring between 1996-2007 and 2008-2020 (OR=0.37, 95% CI 0.37-0.45; P<0.0001). Patients spent a mean of 3.5 days in the ICU, with 86.3% of patients with ‘confirmed OHSS’ requiring at least 2 days of higher level (i.e., level 2 or 3) care. Patients with ‘confirmed OHSS’ required a shorter duration of renal, advanced cardiovascular, and advanced respiratory support than patients ‘without OHSS’ (P<0.0001 for all comparisons). There was no significant difference in BMI or ethnicity between those with ‘confirmed OHSS’ and those ‘without OHSS’, however women with ‘confirmed OHSS’ were younger (34 versus 41 years old, p<0.0001).

**Discussion:**

Although absolute rates of OHSS requiring ICU admission recorded in this study are likely to represent a significant underestimate of all clinically significant OHSS, rates of OHSS requiring ICU admission have decreased since 1996 in concordance with the introduction of modern IVF practices.

## Introduction

Assisted reproductive technology (ART), such as *in vitro* fertilization (IVF), offers people suffering with infertility the opportunity to conceive resulting in the birth of more than eight million children world-wide (1, 2). The most serious complication of IVF treatment is ‘Ovarian Hyperstimulation Syndrome’ (OHSS), which predominantly occurs due to the use of human chorionic gonadotropin (hCG) to induce oocyte maturation (2, 3). HCG can cause excessive stimulation of the ovaries and induce the release of inflammatory mediators, most notably, vascular endothelial growth factor (VEGF) (2, 4). VEGF increases vascular permeability leading to leakage of fluid into the third spaces of the body, resulting in ascites, pleural or pericardial effusions, renal failure, electrolyte disturbance, acute respiratory distress syndrome (ARDS), venous thromboembolism, and rarely even death (4, 5). Reported rates of OHSS vary according to the population studied and the criteria used for diagnosis; typically, mild, moderate, and severe OHSS is reported in 33%, 8%, and 2% of IVF cycles, respectively (4, 6). Women with increased *a priori* risk such as those with polycystic ovary syndrome (PCOS), can have ‘clinically significant’ (i.e., moderate to severe) OHSS reported in up to one quarter of IVF cycles (5, 7).

In the United Kingdom (UK), moderate, or more severe forms of OHSS are categorized as ‘B Grade’ incidents (8) and should be reported to the HFEA within 10 working days (9). B Grade incidents are defined as “serious adverse events or reactions”, whilst mild OHSS is a C Grade incident (9). In the United States (US), data from the National Assisted Reproductive Surveillance System (NASS) from 2006 to 2015 showed that moderate OHSS fell from 1.050% to 0.422%, and severe OHSS from 0.383% to 0.106%, respectively (10). The overall decline in OHSS rates observed has been largely attributed to the introduction of modern IVF practices that improve the safety of IVF treatment (2, 5, 11). Such measures include an increased use of GnRH antagonist co-treated protocols from 27.9% to 63.4% cycles between 2004 to 2015 (10, 12), and the use of alternative agents to hCG for triggering oocyte maturation e.g., GnRH agonists (11). Furthermore, there has been increased use of elective single embryo transfer and segmentation, which can attenuate the risk of ‘Late OHSS’ (13, 14). Other changes to IVF protocols include better identification and tailoring of management of women at high-risk of OHSS by using predictive ovarian reserve markers such as anti-Müllerian hormone (AMH) testing, and avoidance of excessive gonadotropin doses during ovarian stimulation (15). Thus, it is possible that these adaptations in IVF practices have reduced the risk of the more severe forms of OHSS over recent years.

Only a small proportion of all clinically significant OHSS requires higher level care with admission to the intensive care unit (ICU), however there is only very limited description in the literature of rates of OHSS requiring ICU care. Thus, to assess the burden of OHSS on the health service, we used a national intensive care registry (ICNARC) to provide an estimate of the trends in clinically significant OHSS rates requiring ICU care between 1996-2020 in England, Wales, and Northern Ireland.

## Methods

### Ethical approval

ICNARC have the approval to hold patient identifiable data under section 251 of the NHS Act 2006 granted by the Confidentiality Advisory Group (CAG) within the Health Research Authority (HRA). The Case Mix Programme (CMP) is an audit of patient outcomes from adult, general critical care units (intensive care and combined intensive care/high dependency units) covering England, Wales, and Northern Ireland. All data collected by the ICNARC is stored, handled, and reviewed annually in line with the HRA and CAG. As per HRA guidelines, additional ethical approval for this study was not required. The approval number for the CMP is PIAG 2-10(f)/2005.

### Data

Data was acquired from the Intensive Care National Audit and Research Centre (ICNARC) database (16), which records data on the characteristics and clinical course of patients admitted to ICU in England, Wales, and Northern Ireland. In the UK, Level 2 care refers to a High Dependency Unit setting providing not more than single organ support usually with one nurse per two patients, whereas Level 3 care refers to an ICU setting providing multi-organ support usually with one-to-one nursing care and a physician always available. These data include the reason for admission to ICU, demographic data, clinical and laboratory findings, and overall outcome of patients during hospital stay between 1996 to 30th September 2020 (**Tables 1**–**3**). Over this period, 431,491 admissions were recorded for women aged between 18-55 years. From these entries, data of 38,957 patients who were either diagnosed with OHSS or were coded with a condition that was of relevance to OHSS were selected (see **Supplementary Table 1** for list of conditions) (16). These initial broad search criteria aimed to identify any patient with a clinical presentation that could be of relevance to the diagnosis of OHSS. However, there was insufficient granularity to enable accurate diagnosis of OHSS in the majority of these patients due to limited recording of additional data such as ‘recent ART’ for example. Therefore, we further restricted the cohort to conditions that are more closely associated with OHSS (18), for example, evidence of thromboembolic disease, pulmonary edema, or acute renal failure (**Supplementary Table 2**). Of these, 120 patients had recent ART treatment but no formal coded diagnosis of OHSS, and were categorized as ‘possible OHSS’.

**Table 1:** Demographic characteristics of patient data retrieved from ICNARC, 1996-2020.

Baseline Characteristics	Without OHSS		Confirmed OHSS			Potential OHSS	
	Count (% of total)	Proportion excluding unavailable data (%)	Count (% of total)	Proportion excluding unavailable data (%)		Count (% of total)	Proportion excluding unavailable data (%)
	**n = 38,599 (99.1%)**		**n = 238** **(0.6%)**			**n = 120** **(0.3%)**	
**Age (years)**									
**Median (IQR)**	41.0 (32.0, 49.0)		34.0 (29.0, 37.8)		p<0.0001	35.0 (31.0, 40.0)	
**< 20**	717 (1.9%)		0 (0.0%)			1 (0.8%)	
**20 to <25**	2592 (6.7%)		14 (5.9%)			8 (6.7%)	
**25 to <30**	3875 (10.0%)		51 (21.4%)			13 (10.8%)	
**30 to <35**	5281 (13.7%)		70 (29.4%)			34 (28.3%)	
**35 to <40**	5300 (13.7%)		62 (26.1%)			33 (27.5%)	
**40 to <45**	4989 (12.9%)		15 (6.3%)			14 (11.7%)	
**45 to <50**	6406 (16.6%)		14 (5.9%)			13 (10.8%)	
**≥ 50**	9439 (24.5%)		12 (5.0%)			4 (3.3%)	
**BMI (kg/m^2^)**									
**Median (IQR)**	26. 1 (22.6, 31.6)		25.5 (22.7, 29.8)		p = 0.17	27.1 (22.9, 31.0)	
**< 20**	2784 (7.2%)	9.7%	9 (3.8%)	7.5%		7(5.8%)	6.9%
**20 to <25**	9484 (24.6%)	33.1%	48 (20.2%)	40.0%		34 (28.3%)	33.7%
**25 to <30**	7438 (19.3%)	25.9%	36 (15.1%)	30.0%		29 (24.2%)	28.7%
**30 to <35**	4156 (10.8%)	14.5%	18 (7.6%)	15.0%		17 (14.2%)	16.8%
**≥ 35**	4825 (12.5%)	16.8%	9 (3.8%)	7.5%		14 (11.7%)	13.9%
**Data Unavailable**	9912 (25.7%)		118 (49.6%)			19(15.8%)	
**Ethnicity**									
**White**	25511 (66.1%)	82.2%	115 (48.3%)	80.4%	p = 0.9	77 (64.2%)	68.1%
**Asian**	2437 (6.3%)	7.9%	16 (6.7%)	11.2%	p = 0.17	17 (14.2%)	15.0%
**Black**	1925 (5.0%)	6.2%	7 (2.9%)	4.9%	p = 0.73	14 (11.7%)	12.4%
**Mixed Ethnicities**	430 (1.1%)	1.4%	3 (1.3%)	2.1%	p = 0.46	1 (0.8%)	0.9%
**Undefined Ethnic Group**	721 (1.9%)	2.3%	2 (0.8%)	1.4%	p = 0.78	4 (3.3%)	3.5%
**Data unavailable**	7575 (19.6%)		95 (39.9%)			7 (5.8%)	

Demographic data of female patients aged between 18-55 years presenting to an ICU in England, Wales or Northern Ireland. Data is collected from the ICNARC database (17) presented between the years 1996 to September 30^th^, 2020.

Data are presented as median (25^th^ quartile, 75^th^ quartile). A percentage proportion (%) is shown for patients within each category including and excluding unavailable data. Values are presented prior to any normalization process.

Ethnic groups presented include the following: White: British, Irish or Any Other White; Asian: Indian, Pakistani, Bangladeshi, Chinese or Any Other Asian; Black: Caribbean, African or Any Other Black.

**^†^** Data are presented as the count of patients, followed by the proportion of patients within each category (%).

*Patients ‘without OHSS’ have been compared to those with ‘confirmed OHSS’ by Mann-Whitney U testing (for Age and Body Mass Index), and Fisher’s Exact testing (for Ethnicity) to generate a P value, presented to 2 decimal places where appropriate. p values presented in **bold** represent statistically significant differences (p<0.05).

ICNARC, Intensive Care National Audit & Research Centre; OHSS, Ovarian Hyperstimulation Syndrome; IQR, Interquartile Range; BMI, Body Mass Index; ICU, Intensive Care Unit.

**Table 2:** Laboratory and clinical findings of patients requested from ICNARC, 1996-2020.

Clinical Characteristics	Without OHSS	Confirmed OHSS	P-value	Potential OHSS
Median (IQR)	n = 38599	n = 238		n = 120
**Lowest Systolic Blood Pressure (mmHg)**	92.0 (82.0, 105.0)	105.0 (95.5, 115.0)	**p < 0.0001**	100.0 (90.0, 111.0)
**Highest Systolic Blood Pressure (mmHg)**	140.0 (125.0, 156.0)	142.0 (130.0, 155.0)	p = 0.11	147.0 (131.5, 162.0)
**Lowest Heart Rate (bpm)**	75.0 (64.0, 89.0)	82.0 (72.0, 95.0)	**p < 0.0001**	71.0 (63.5, 84.0)
**Highest Heart Rate (bpm)**	110.0 (95.0, 125.0)	113.0 (100.0, 125.0)	p = 0.02	104.0 (90.0, 115.5)
**Lowest Non-Ventilated Respiratory Rate (bpm)**	13.0 (10.0, 16.0)	14.0 (12.0, 17.0)	**p < 0.0001**	13.0 (11.0, 15.0)
**Highest Non-Ventilated Respiratory Rate (bpm)**	24.0 (20.0, 30.0)	26.0 (22.0, 30.0)	**p = 0.009**	22.0 (20.0, 27.0)
**Lowest Serum Creatinine (pmol/L)**	65.0 (50.0, 107.0)	61.0 (51.0, 76.0)	**p = 0.005**	67.5 (51.3, 111.8)
**Highest Serum Creatinine (pmol/L)**	78.0 (58.0, 146.0)	67.0 (59.0, 84.3)	**p = 0.0003**	87.0 (62.0, 126.0)
**Highest Serum Urea (mmol/l_)**	5.1 (3.4, 9.4)	4.4 (3.4, 6.1)	**p < 0.0001**	5.6 (3.4, 9.9)
**Lowest Serum Potassium (mmol/L)**	3.9 (3.5, 4.3)	4.1 (3.8, 4.5)	**p < 0.0001**	4.1 (3.8, 4 5)
**Highest Serum Potassium (mmol/L)**	4.5 (4.1, 5.0)	4.6 (4.2, 5.0)	p = 0.30	4.7 (4.2, 5.2)
**Lowest Serum Sodium (mmol/L)**	136.0 (133.0, 139.0)	132.0 (128.0, 136.0)	**p < 0.0001**	136.0 (133.0, 138.0)
**Highest Serum Sodium (mmol/L)**	139.0 (137.0, 142.0)	134.0 (130.0, 138.0)	**p < 0.0001**	139.0 (136.0, 142.0)
**Highest Blood Lactate (mmol/L)**	1.9 (1.2, 3.4)	1.3 (0.9, 1.7)	**p < 0.0001**	2.1 (1.5, 3.1)

Laboratory and clinical findings of female patients aged between 18-55 years presenting to an ICU in England, Wales, or Northern Ireland. The ICNARC database (17) does not collect the ‘lowest’ and ‘highest’ value recorded for all observational parameters. The medians and IQRs of these datasets have been presented. Data is presented between the years 1996 to September 30th, 2020. Data are presented as median (25th quartile, 75th quartile).

*P values were generated from Mann-Whitney U tests between patients ‘without OHSS’ and those with ‘confirmed OHSS’. P values presented in bold represent statistically significant differences (P<0.05).

ICNARC, Intensive Care National Audit & Research Centre; OHSS, Ovarian Hyperstimulation Syndrome; IQR, Interquartile Range; BP, Blood Pressure; ICU, Intensive Care Unit.

**Table 3:** Care and support needs for patients retrieved from ICNARC between 1996-2020.

ICU Care	Without OHSS		Confirmed OHSS			Potential OHSS
	Count (% of total)	Proportion (excluding unavailable data) (%)	Count (% of total)	Proportion (excluding unavailable data) (%)		Count (% of total)
**Days of Renal Support** **Mean (SD)**	**0.80 (± 3.4)**		**0.03 (± 0.41)**			**0.0 (± 0.0)**
0	27,996 (72.5%)	86.30%	145 (60.9%)	99.32%		111 (92.5%)
>0	4431 (11.5%)	13.70%	1 (0.4%)	0.68%		9 (7.5%)
Data Unavailable	6172 (16.0%)		92 (38.7%)		p<0.0001	0 (0.0%)
**Days of Cardiovascular Support** **Mean (SD)**	**0.62 (± 2.0)**		**0.10 (± 0.43)**			**0.23 (± 0.63)**
0	25368 (65.7%)	78.20%	138 (58.0%)	99.32%		104 (86.7%)
>0	7059 (18.3%)	21.80%	8 (3.4%)	0.68%		16 (13.3%)
Data Unavailable	6172 (16.0%)		92 (38.7%)		p<0.0001	0 (0.0)%
**Days of Respiratory Support** **Mean (SD)**	**2.5 (± 6.8)**		**0.15 (± 0.76)**			**1.52 (± 5.29)**
0	18049 (46.8%)	55.70%	137 (57.6%)	93.84%		67 (55.8%)
>0	14378 (37.2%)	44.30%	9 (3.8%)	6.16%		53 (44.2%)
Data Unavailable	6172 (16.0%)		92 (38.7%)		p<0.0001	0 (0.0%)
**Days of Level 0 Care** **Mean (SD)**	**0.05 (± 0.4)**		**0.05 (± 0.4)**			**0.02 (± 0.1)**
0	31539 (81.7%)	97.30%	142 (59.7%)	97.26%		118 (98.3%)
1	622 (16%)	1.90%	3 (1.3%)	2.05%		2 (1.7%)
2	145 (0.4%)	0.40%	0 (0.0%)	0.00%		0 (0.0%)
3	55 (0.1%)	0.20%	0 (0.0%)	0.00%		0 (0.0%)
4	30 (0.1%)	0.10%	1 (0.4%)	0.68%		0 (0.0%)
>4	34 0.1%	0.10%	0 (0.0%)	0.00%		0 0.0%
Data Unavailable	6174 (16.0%)		92 (38.7%)		P = 0.99	0 (0.0%)
**Days of Level 1 Care** **Mean (SD)**	**0.26 (± 0.90)**		**0.45 (± 1.01)**			**0.25 (± 0.84)**
0	27719 (71.8%)	85.50%	112 (47.1%)	76.71%		0 (0.0%)
1	2986 (7.7%)	9.20%	16 (6.7%)	10.96%		107 (89.2%)
2	938 (2.4%)	2.90%	12 (5.0%)	8.22%		5 (4.2%)
3	370 (1.0%)	1.10%	3 (1.3%)	2.05%		3 (2.5%)
4	175 (0.5%)	0.50%	0 (0.0%)	0.00%		2 (1.7%)
>4	239 (0.6%)	0.70%	3 1 3%	2.05%		2 (1.7%)
Data Unavailable	6172 (16.0%)		92 (38.7%)		p=0.002	1 (0.8%)
**Days of Level 2 or 3 Care** **Mean (SD)**	**5.2 (± 8.1)**		**3.5 (± 0.9)**			**1.8 (± 5.5)**
0	312 (0.8%)	1.00%	4 (1.7%)	2.74%		3 (2.5%)
1	3851 (10.0%)	11.90%	16 (6.7%)	10.96%		19 (15.8%)
2	10389 (26.9%)	32.00%	43 (18.1%)	29.45%		58 (48.3%)
3	5313 (13.8%)	16.40%	29 (12.2%)	19.86%		18 (15.0%)
4	3061 (7.9%)	9.40%	17 (7.1%)	11.64%		8 (6.7%)
>4	9501 (24.6%)	29.30%	37 (15.6%)	25.34%		14 (11.7%)
Data Unavailable	6172 (16.0%)				P = 0.32	0 0.0%
**APACHE II Score** **Median (IQR)**	**12.0 (9.0, 18.0)**		**8.0 (5.3, 11.0)**			**10.0 (7.0, 13.0)**
<15	23610 (61.2%)		219 (92.0%)			8 (81.7%)
15 - 24	11460 (29.7%)		18 (7.6%)			20 (16.7%)
25 - 35	3039 (7.9%)		1 (0.4%)			2 (1.7%)
>35	490 (1.3%)		0 (0.0%)			0 (0.0%)
Data Unavailable	0 (0.0%)		0 (0.0%)		p<0.0001	0 (0.0%)

Care and support needs of female patients aged between 18-55 years presenting to an ICU in England, Wales, or Northern Ireland. Data is presented between the years 1996 to September 2020. Data are presented as median (25^th^ quartile, 75^th^ quartile). A percentage proportion (%) is shown for patients within each category including and excluding unavailable data (there was no missing data for patients with ‘potential OHSS’, so this column has been excluded).

**†**Data are presented as the count of patients, followed by the proportion of patients within each category (%).

*Patients ‘without OHSS’ have been compared to those with ‘confirmed OHSS’ by Mann-Whitney U testing) to generate a P value, presented to 2 decimal places. P values presented in **bold** represent statistically significant differences (P<0.05).

ICNARC, Intensive Care National Audit & Research Centre; OHSS, Ovarian Hyperstimulation Syndrome; APACHE II Score, Acute Physiology and Chronic Health Evaluation II Score; ICU, Intensive Care Unit.

### Data categorization

The retrieved 38,957 patients were categorized into three groups (**Tables 1**–**3**). Patients with OHSS recorded as the reason for ICU admission were classified as ‘confirmed OHSS’ cases (n=238). Patients diagnosed with a condition possibly related to OHSS in addition to a history of recent assisted reproductive technology (ART) were classified as ‘potential OHSS’ cases (n=120, **Supplementary Table 2** for full list of OHSS-related conditions). All other patients were classified as ‘without OHSS’ (n=38,599). We did not include ‘potential OHSS’ cases in analyses of OHSS cases as we could not be certain that their presentation was due to OHSS. Therefore, statistical comparisons were restricted to comparison of patients with ‘confirmed OHSS’ to those ‘without OHSS’.

The numbers of ‘confirmed’ and ‘potential’ OHSS cases in 2020 were proportionately adjusted to account for the lack of availability of data between 1st October 2020 and 31st December 2020 at time of data-extraction. The number of ICUs from which the ICNARC database (17) collects data has increased significantly in recent years to now include all centres in England, Wales, and Northern Ireland (n=286; **Supplementary Figure 1**). Thus, a proportional uplift in the number of cases (termed ‘normalized’) according to the number of reporting centres was applied to account for lower number of ICUs reporting to ICNARC in the earlier years of the database. The rate of OHSS was expressed as a proportion of the number of ‘confirmed’ OHSS cases after normalization and the number of fresh IVF cycles conducted each year as reported by the HFEA in England, Wales, and Northern Ireland (since the ICNARC database does not include data from Scotland) (16, 19).

### Statistical analysis

Demographic and clinical data were presented descriptively as count with proportion (%) for categorical data, and median (IQR) for continuous data, as appropriate. Demographic and clinical data was compared between patients ‘without OHSS’, and those with ‘confirmed OHSS’ by the Mann Whitney U test, or Fisher’s exact test, as appropriate. The Mann-Kendall test was used to assess the overall trend in OHSS rates from 1996 to 2018 (18) (19). Microsoft Excel and GraphPad Prism version 9.0 were used for data presentation and statistical analysis. The statistical significance level was set at 0.05 for all analyses.

## Results

A total of 38,957 female patients aged 18-55 years from 1996 to September 30th 2020, were retrieved from the ICNARC database. Of these, 238 (0.61%) women were classified as having ‘confirmed OHSS’, a further 120 (0.31%) having ‘potential OHSS’, and 38,599 (99.1%) as being ‘without OHSS’ (**Table 1**). The majority of women in all three groups were Caucasian and had a BMI under 30 kg/m^2^. Women with ‘confirmed OHSS’ were younger than those ‘without OHSS’ (34 vs 41 years; P<0.0001, **Table 1**), but no significant differences in body mass index (BMI) (P=0.17) or ethnicity were observed (**Table 1**).

### Prevalence and incidence of OHSS

**Figure 1:**
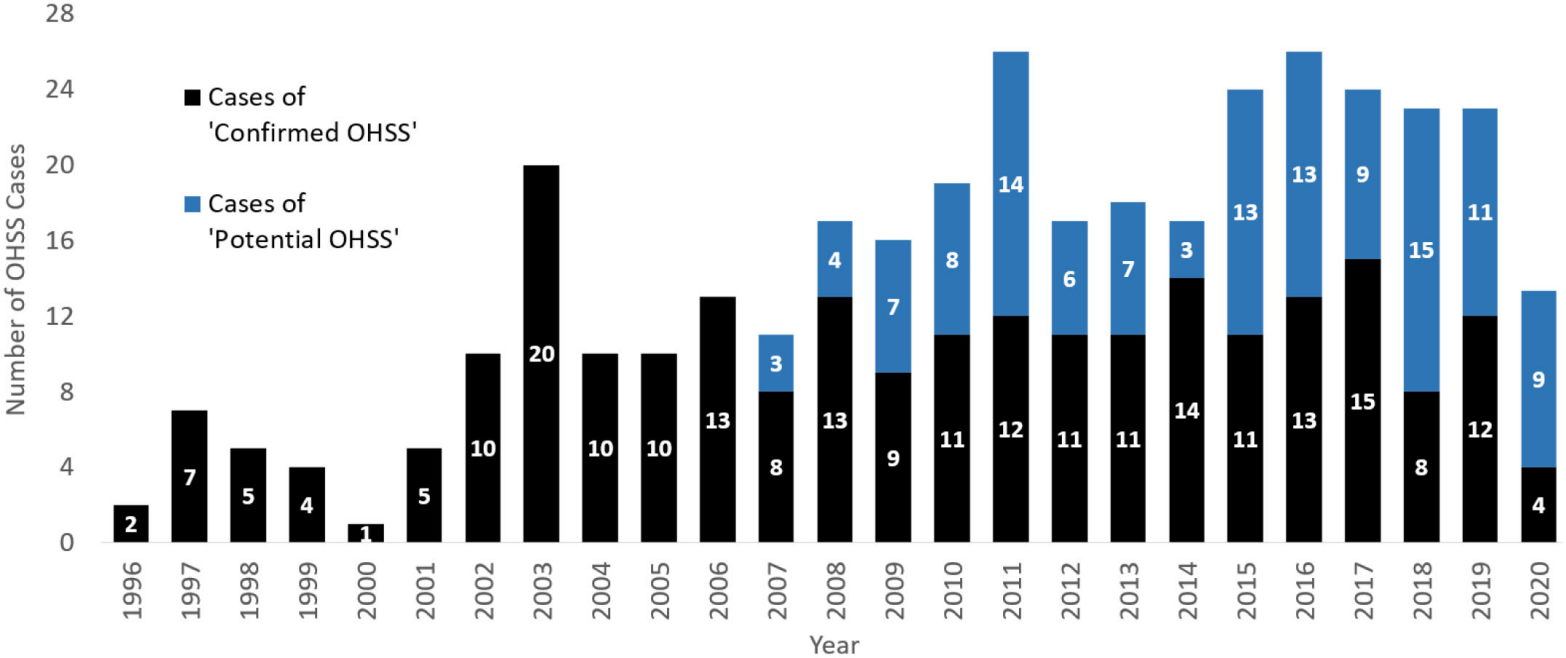
The absolute number of ‘confirmed’ and ‘potential’ OHSS cases from 1996-2020. The annual combined number of ‘confirmed’ and ‘potential’ OHSS cases between 1996 to September 2020. ‘Potential OHSS’ includes patients who presented to ICU with an OHSS-related condition and had a history of recent ART. All female patients were aged between 18-55 years presenting to an ICU in England, Wales, or Northern Ireland. OHSS cases for the year 2020 have been adjusted to account for the lack of data between October-December 2020. OHSS, Ovarian Hyperstimulation Syndrome; ICU, Intensive Care Unit; ART, Assisted Reproductive Technology.

**Figure 2:**
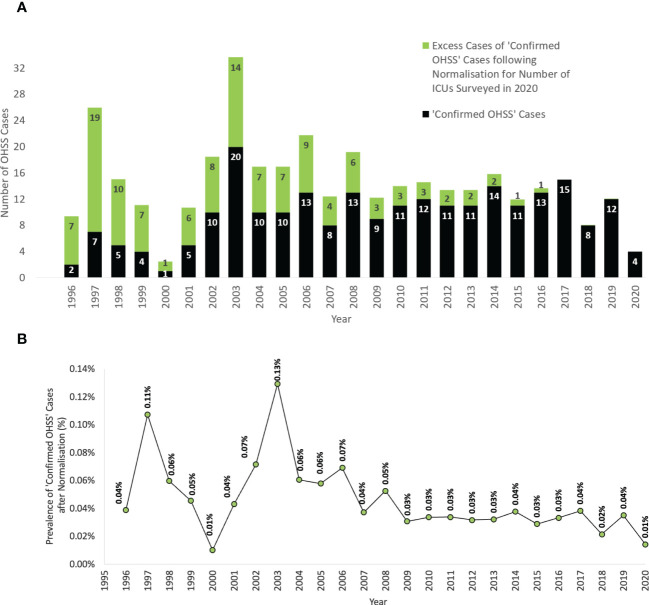
The rate of ‘confirmed OHSS’ cases following normalization for the number of reporting centers from 1996-2018. **(A)** The total absolute number of ‘confirmed OHSS’ cases before normalization. **(B)** The prevalence (%) of ‘confirmed OHSS’ cases expected after normalization for the number of ICUs surveyed in 2020, expressed as a proportion of the number of fresh IVF cycles per year in England, Wales, and Northern Ireland. All female patients were aged between 18-55 years presenting to an ICU in England, Wales, or Northern Ireland. OHSS, Ovarian Hyperstimulation Syndrome; ICU, Intensive Care Unit; IVF, *In Vitro* Fertilization.

The annual number of ‘confirmed OHSS’ cases appeared to be greater in recent years, and this trend is exaggerated when these figures are combined with the number of ‘potential OHSS’ cases (**Figure 1**). However, as the number of ICUs reporting to the ICNARC database also generally increased over this time-period, when the annual numbers of ‘confirmed OHSS’ cases were normalized in proportion to the number of ICUs reporting to the ICNARC database in 2020 (**Supplementary Figure 1**), the annual number of ‘confirmed OHSS’ cases decreased over recent years (**Figure 2A**).

The number of fresh IVF cycles have also increased each year. In order to evaluate the rate of OHSS requiring ICU admission per fresh IVF cycle, the number of cases were expressed as a proportion of the annual number of IVF cycles conducted in England, Wales, and Northern Ireland (1). This proportion decreased between 1996-2018 (P=0.006, Mann-Kendall Test, **Figure 2B**), with the average rate of ‘confirmed OHSS’ requiring ICU admission from 2008-2020 (0.03% ≈ 1 in 3125) being significantly lower than when compared to that between 1996-2007 (0.06% ≈ 1 in 1750) [P<0.0001, Fisher’s Exact test, OR=0.37, 95% CI 0.37-0.45; P<0.0001].

### Clinical and laboratory data of OHSS

Clinical and laboratory data for patients on admission to ICU with ‘confirmed’ and ‘without OHSS’ are presented in **Table 2**. The ‘lowest systolic blood pressure’ in patients with ‘confirmed OHSS’ was higher than in patients ‘without OHSS’ (P<0.0001), however there was no evidence for a statistically significant difference in the ‘highest systolic blood pressure’ between the groups (P=0.11). Patients with ‘confirmed OHSS’, had higher heart rate (both lowest recorded heart rate, P<0.0001; and highest recorded heart rate, P=0.02) and increased non-ventilated respiratory rate (lowest non-ventilated, P<0.0001; highest non-ventilated, P=0.009) compared to patients ‘without OHSS’.

Both serum creatinine (lowest serum creatinine, P=0.005; highest serum creatinine, P=0.0003) and urea (P<0.0001) were lower in patients with ‘confirmed OHSS’ and these women were also less likely to have an elevated blood lactate (P<0.0001). Patients with ‘confirmed OHSS’ had a lower serum sodium level than patients ‘without OHSS’ (P<0.0001). Notably, the proportion of patients with their ‘lowest serum sodium’ value signifying hyponatremia (i.e., below 135mmol/L) was almost 2-fold greater in the ‘confirmed OHSS’ group compared to the ‘without OHSS’ group (65.6%, 34.5%; P<0.0001).

### ICU support data for OHSS

**Figure 3:**
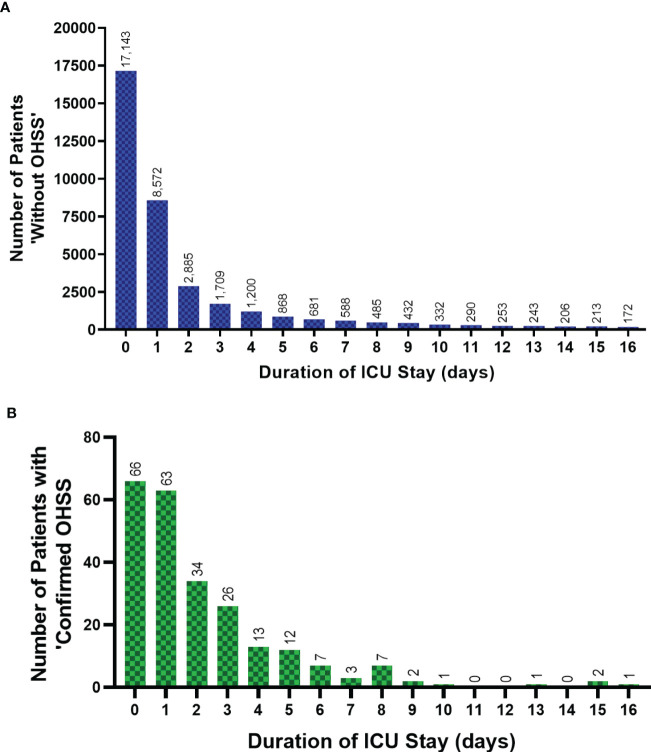
The ICU duration of stay for patients ‘without OHSS’ and ‘confirmed OHSS’ cases from 1996-2020. The ICU length of stay (up to 16 days) of female patients aged 18-55 years in England, Wales and Northern Ireland, 1996-Sept 2020, showing **(A)** patients ‘without OHSS’, and **(B)** patients with ‘confirmed OHSS’. Two outliers, ‘783’ and ‘2557’ days not included for two patients ‘without OHSS’. Data between 17-553 days spent in ICU (n=2,331 patients) for patients ‘without OHSS’ has not been presented in Figure 3A to allow its direct comparison with Figure 3B. Data is presented without normalization or adjustment. Coverage of ICUs in England, Wales and Northern Ireland has increased over time and therefore, relevant historical admissions for OHSS or potential OHSS are likely to be excluded. ICU, Intensive Care Unit; OHSS, Ovarian Hyperstimulation Syndrome.

Care and support needs of patients ‘without OHSS’, and those with ‘confirmed OHSS’ are presented in **Table 3**. Patients with ‘confirmed OHSS’ required a shorter duration of renal, advanced cardiovascular, and advanced respiratory support, than patients ‘without OHSS’ (P<0.0001 for all comparisons). The mean length of level 1 care stay was significantly higher in women with ‘confirmed OHSS’ than in those without (0.45 days versus 0.26 days, P=0.002; **Table 3**) (**Figure 3**). Patients with ‘confirmed OHSS’ spent a mean of 3.5 days compared with 5.2 days for those ‘without OHSS’ in level 2-3 care (patients requiring more detailed observation and intervention; (P=0.32), with 86.3% of patients with ‘confirmed OHSS’ requiring at least 2 days of care at these levels. The Acute Physiology and Chronic Health Evaluation II score (APACHE II) score classifies the severity of a patient’s condition in critical care units, with higher scores denoting more severe disease. Patients with ‘confirmed OHSS’ had lower APACHE II scores than those ‘without OHSS’ (8 vs 12; P<0.0001).

## Discussion

This is the first study to demonstrate that the rates of OHSS requiring ICU admission have decreased over the past two decades in England, Wales, and Northern Ireland. There are very limited published reports on the rates of OHSS requiring ICU admission, and although it is mandatory to report OHSS cases requiring ICU admission in Europe, this is not a requirement in the US (20).

The rate of ‘confirmed OHSS’ requiring ICU admission per fresh IVF cycle has halved over recent years, from 0.06% in 1996-2006 to 0.03% in 2007-2019. This decline is likely to reflect the introduction of safer IVF practices including individualized dosing of gonadotropins for ovarian stimulation, using predictive markers such as AMH (15, 21), and increased use of GnRH antagonist co-treated protocols (12, 17). Furthermore, the reduced use of fresh embryo transfer cycles (13, 14) from 82% in 2008 to 77% in 2019 (19), and the increased use of frozen embryo transfer (FET) may also have contributed to a reduction in OHSS requiring ICU admission (13, 22, 23). Although the segmentation of fresh and frozen cycles does not affect the risk of ‘Early OHSS’ (14, 24) it can attenuate the risk of ‘Late OHSS’ which is more challenging to manage (25). While the use of FET cycles is increasing (from 10% in 1991 to 41% in 2019), fresh transfers are still conducted in the majority of IVF cycles in the UK (19, 22).

The true impact of such prevention strategies on reducing the incidence of all clinically significant OHSS remains unclear (10, 13, 22) and is likely reflective of the challenges in accurately reporting OHSS. For example, the current diagnostic criteria lack specificity (e.g. the precise threshold for the volume of ascites on ultrasound to signify ‘moderate’ OHSS is not clearly defined) and are derived based on historic studies using older technologies. Additionally, assessment by ultrasound and blood examination is required in order to make a diagnosis of moderate or more severe forms of OHSS, which is not routinely conducted. Thus, the true rates of OHSS cases could be markedly underestimated (4, 26). For example, from 2015 to 2016, there were 865 admissions to hospital in the UK (of which, 836 were emergency admissions) for whom OHSS was the presenting complaint, however, only 98 serious cases of OHSS were reported to the HFEA, which would have excluded patients who presented directly to their reproductive medicine unit during working hours (26).

In line with this data, moderate to severe OHSS rates in the United States have halved from 1.2% in 2000-2006 to 0.5% in 2006-2015 (10). Data from France between 2012-2017 reported that OHSS accounted for 61% of IVF-related complications, with 1.6% of oocyte retrievals resulting in the need for OHSS-related hospital admission (27). However, these rates are higher than those observed in the present study as they report all cases of moderate to severe OHSS, not just those requiring ICU admission. Furthermore, the number of cases in the current report could be under-represented as coding of a history of recent ART in ICNARC only became routine after 2007 (only 11% of admissions reported this admission prior to 2007). Thus, it is important to note that most cases of mild to moderate OHSS are unlikely to be captured in the current dataset, as most patients can be managed in the outpatient setting (20, 28). Although some countries such as the UK, Ireland, and Australia (18, 20, 28) solely recommend inpatient treatment for severe OHSS, the American Society for Reproductive Medicine (ASRM) (29) and Canadian Guidelines (30) acknowledge the role of outpatient management in select cases of severe OHSS. Nevertheless, most cases of severe OHSS do not require higher level care in ICU even if hospital admission is indicated. Notably, the Office for National Statistics reported two deaths directly due to OHSS in England and Wales between 2005-2006 (31), however these patients were not apparent in the ICNARC database, suggesting that not all patients with life-threatening OHSS have been captured in the ICNARC database.

The present study found that the majority of patients who had ‘confirmed OHSS’ were under the age of 35 years, which could partly be explained by greater accessibility of IVF treatment for these women in the UK (32). Furthermore, younger women have a greater ovarian response to ovarian stimulation than older women, such that younger age is an established risk factor for OHSS (33, 34). Although low BMI has been regarded as a risk factor for OHSS, our study did not demonstrate a significant difference in BMI between patients with ‘confirmed OHSS’ and those ‘without OHSS’, and this has also been confirmed by more recent studies (25, 33, 35). This difference could also have been due to inadequate dose-adjustment of FSH for body weight in previous years (36, 37). With regards to ethnicity, 78% of IVF cycles in the UK are carried out in ‘White’ women (38), which reflects our findings, with the majority of patients with ‘confirmed OHSS’ being of ‘White’ ethnicity (39). Women of ‘Black’ ethnic backgrounds have been reported to be more likely to develop moderate-severe OHSS compared to their ‘White’ counterparts potentially due to FSH-receptor polymorphisms (40). However, the present study did not demonstrate any relationship between ethnicity, frequency of IVF cycles and OHSS requiring ICU admission. Women with or without OHSS both required similar duration of treatment in ICU, despite the former being younger and potentially less likely to have co-morbidities.

Hyponatremia occurs in clinically significant OHSS (41, 42), likely due to secondary hyperaldosteronism resulting in fluid retention and inappropriate anti-diuretic hormone (ADH) secretion due to intravascular depletion (43, 44). In rat models of OHSS, inhibition of ADH led to a decreased risk of OHSS (45). This study found that 66% of patients with ‘confirmed OHSS’ had a serum sodium of <135 mmol/L, whilst 7% had a serum sodium of <125 mmol/L, consistent with intravascular hemodilution. Additionally, secondary hyperaldosteronism would be expected to result in hypokalemia, however, OHSS was more commonly associated with hyperkalemia in the present dataset. One quarter of patients with ‘confirmed OHSS’ had hyperkalemia but only 34% of these patients had evidence of renal impairment to explain this. This percentage is higher than expected in those undergoing routine ART as it relates to patients requiring admission to ICU. Thus, it is possible that other processes resulting in hyperkalemia could have been present such as cell-death (46). Based on serum creatinine, this study found that only 15% of ‘confirmed OHSS’ had renal dysfunction classified by ICNARC as “severe” (serum creatinine level of 88-133 μmol/L), whilst 10% were “life-threatening” (serum creatinine levels greater than 133 μmol/L) (47). This suggests that either renal dysfunction is an infrequent occurrence in patients with OHSS requiring ICU admission, or that the presence of intravascular hemodilution (as suggested by hyponatremia) could lead to a falsely reassuring creatinine level (18, 47).

Limitations of this study include the need for normalization for the number of reporting centres and number of fresh IVF cycles conducted over the last two decades (16). Furthermore, data on the detail of ART protocols conducted, and other OHSS parameters such as ascitic volume was not available. It is also likely that the number of cases of severe OHSS has been underestimated, given that two known deaths due to OHSS from the Office of National Statistics (ONS) (31) covering the same time-period were not captured in the ICNARC database.

## Conclusion

Rates of clinically severe OHSS requiring ICU admission in the UK have decreased over the past two decades. Due to its focus on OHSS requiring ICU admission, this study likely presents the absolute minimum prevalence of severe OHSS and thus is likely to underestimate the occurrence of all clinically significant OHSS. Although rates of OHSS have reduced, they have not been eliminated, and improved IVF practices including individualized approaches should be continued in order to minimize the residual risk of this serious iatrogenic complication of IVF treatment.

## Data availability statement

The raw data supporting the conclusions of this article will be made available by the authors, without undue reservation.

## Ethics statement

ICNARC have the approval to hold patient identifiable data under section 251 of the NHS Act 2006 granted by the Confidentiality Advisory Group (CAG) within the Health Research Authority (HRA). The Case Mix Programme (CMP) is an audit of patient outcomes from adult, general critical care units (intensive care and combined intensive care/high dependency units) covering England, Wales, and Northern Ireland. All data collected by the ICNARC is stored, handled, and reviewed annually in line with the HRA and CAG. As per HRA guidelines, additional ethical approval for this study was not required. The approval number for the CMP is PIAG 2-10(f)/2005. Written informed consent for participation was not required for this study in accordance with the national legislation and the institutional requirements.

## Author contributions

All authors have contributed to the study and the development of the manuscript.
