# Supplementary material for: Ovarian Hyperstimulation Syndrome (OHSS) requiring Intensive Care Unit (ICU) admission between 1996-2020 in England, Wales, and Northern Ireland
Source: Front Endocrinol (Lausanne). 2022 Dec 15;13:1060173. doi: 10.3389/fendo.2022.1060173 (PMC9797661; doi:10.3389/fendo.2022.1060173)
Supplement: Supplementary file 1 [file DataSheet_1.docx]

**Supplementary Table 1: Search criteria for OHSS and related conditions from the ICNARC Database**


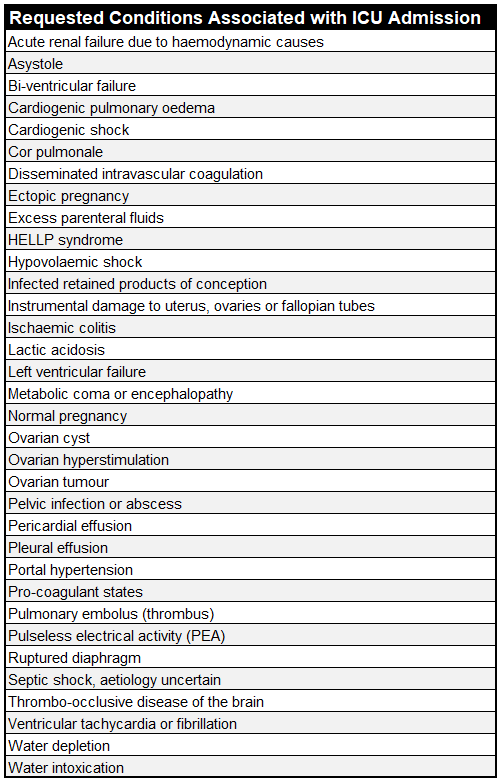


The search criteria of conditions associated with patient admission to ICU, requested from the ICNARC database. The demographic and clinical data of patients with any of these condition as a reason for admission to ICU were retrieved to analyse ‘confirmed’ and ‘potential’ cases of OHSS.

**Supplementary Table 2: OHSS-related reasons for ICU admission, 1996-2020.**

| Reason for Admission to ICU | Number of Patients with Each Condition | | |
| --- | --- | --- | --- |
|  | **Patients with History of Recent ART †** | | **Patients with Unknown Recent ART Status** |
| Acute renal failure due to haemodynamic causes | 13 | 2,031 | |
| Bi-ventricular failure | 2 | 59 | |
| Cardiogenic pulmonary oedema | 4 | 118 | |
| Cardiogenic shock | 1 | 91 | |
| Disseminated intravascular coagulation | 2 | 10 | |
| HELLP syndrome | 40 | 444 | |
| Hypovolaemic shock | 35 | 2,903 | |
| Lactic acidosis | 4 | 72 | |
| Left ventricular failure | 1 | 49 | |
| Pro-coagulant states | 1 | 5 | |
| Pulmonary embolus (thrombus) | 6 | 799 | |
| Septic shock, aetiology uncertain | 10 | 480 | |
| Thrombo-occlusive disease of brain | 1 | 26 | |
| Total | **120** | **7,087** | |

The ART status of patients with OHSS-related reasons for ICU admission. Data include female patients aged between 18-55 years presenting to an ICU in England, Wales, or Northern Ireland. Data is provided between the years 1996 to September 30^th^, 2020.

**^†^** ‘Potential OHSS’ refers to a patient who had a condition related to OHSS along with a history of recent ART.

ART, Assisted Reproductive Technology; OHSS, Ovarian Hyperstimulation Syndrome; ICU, Intensive Care Unit; HELLP syndrome, Haemolysis Elevated Liver enzymes and Low Platelet count syndrome of pregnancy.

**Supplementary Figure 1: The annual number of ICUs reporting to ICNARC from 1996-2020.**


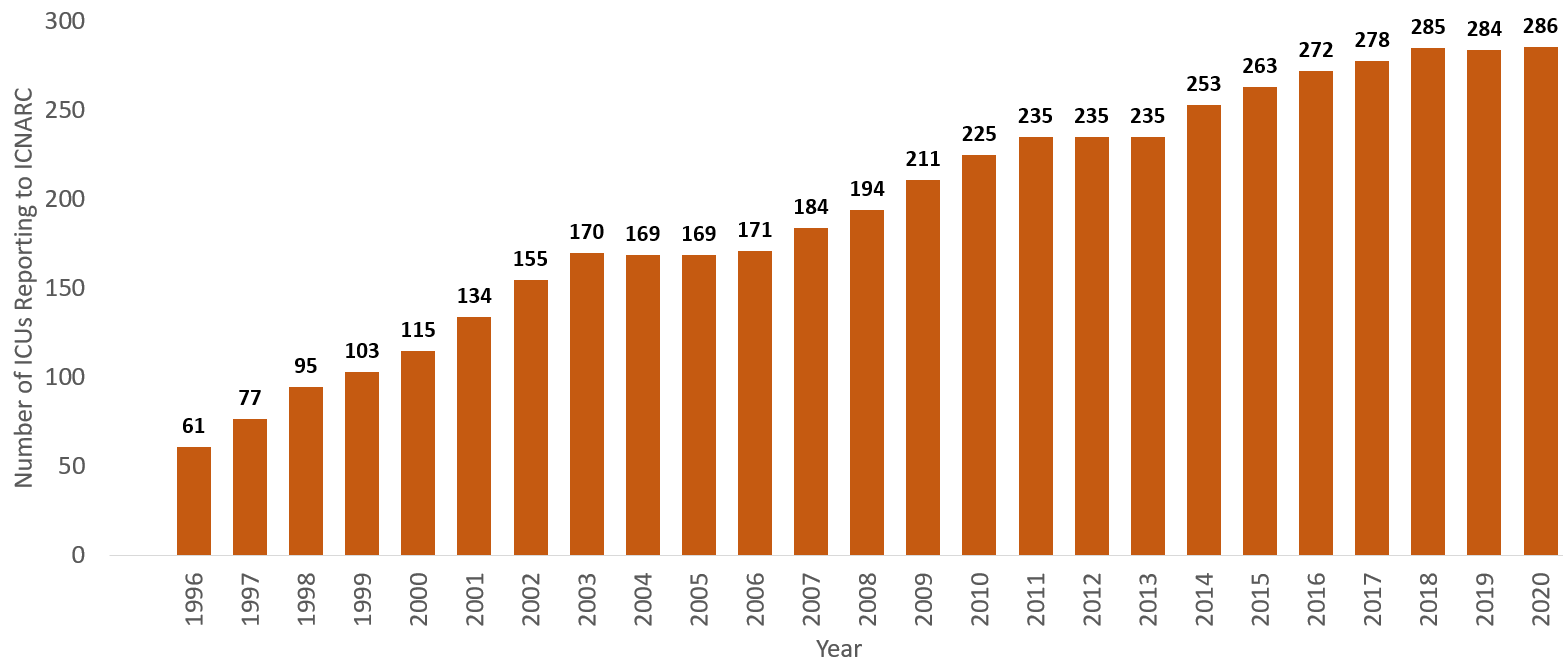


The number of ICUs in England, Wales and Northern Ireland reporting at least one admission of a female patient that met the search criteria between the ages of 18-55 years to the ICNARC database. Data is presented between the years 1996 to September 2020.

OHSS, Ovarian Hyperstimulation Syndrome; ICNARC, Intensive Care National Audit & Research Centre; ICU, Intensive Care Unit.
